# Recruiting medical students from underrepresented backgrounds to a project to identify support challenges amongst their peers whilst encouraging early career engagement in psychiatry

**DOI:** 10.1192/bjo.2021.390

**Published:** 2021-06-18

**Authors:** Heather Gail McAdam, Debbie Aitken

**Affiliations:** 1 University of Edinburgh; 2 University of Edinburgh, University of Cambridge

## Abstract

**Aims:**

To engage lived experience individuals to run a project identifying the mental health challenges unique to medical students who self identify as belonging to marginalised groups;

To use the project findings to inform mental health support and education during medical training and beyond;

To encourage the individuals to engaged mental health policy and education whilst also using the process to inform their future medical careers, including in the field of psychiatry.

**Method:**

Lived experience individuals were recruited to the project following open applications from medical students. The role was to design a project to create an evidence base for tailored support methods needed to reduce marginalised medical students’ increased risk of poor mental health. This formed the basis of guidance on how medical schools and healthcare systems can improve wellbeing support for their students and staff. With support from a trained staff and student member, the recruited officers were encouraged to follow their preferred method for fulfilling the project aims, using their own and peers’ experiences to inform what was most useful.

**Result:**

Representatives were selected from BAME, LGBT+, international, disabled and widening participation backgrounds. The students decided to conduct a survey open to all medical student colleagues across the United Kingdom. The survey questions were split into four sections based on the challenges faced by their own lived experience: General Information; University and Community Experiences; Medical School Experiences and Teaching and Clinical Experiences. There were 58 questions in total including 26 multiple choice; 24 open answers; and 8 Likert scale.

Following data collection, the information taken from the survey and focus groups, supported by background reading, was thematically analysed to identify the key challenges. This will then be used to create a report to share with the medical school containing areas for improvement in mental health support, education and engagement. The officers themselves would also reflect on their experiences throughout the process, including their ability to engage in mental health policy, education and further career options such as psychiatry.

**Conclusion:**

From creating an appropriate and supportive structure, it can be possible to encourage students with lived experience to share their challenges whilst becoming engaged in mental health policy and support. Furthermore, from creating a culture of reflection in the area of mental health, they are helping raise awareness of the subject early on in medical careers and promote engagement into specialties such as psychiatry.

